# *To Pace Or Not To Pace!* — Prevention Of Atrial Fibrillation After Coronary Artery Bypass Surgery

**Published:** 2005-01-01

**Authors:** Amit Vora

**Affiliations:** Consultant cardiologist and Electrophysiologist, Mumbai

Atrial fibrillation (AF) is a very undesirable, but unfortunately a common arrhythmia following coronary artery bypass graft (CABG) surgery, occurring in up to 40% of patients [1]. There is an increase in hospital stay [2] and adds to the overall cost of the surgery. Atrial fibrillation occurrence may identify a subset of patients with reduced survival [3]. Prevention of AF therefore would have a significant positive impact on patients undergoing CABG surgery.

The pathophysiology of post-operative AF is incompletely understood and appears multi-factorial. There are studies to suggest clinical factors like advanced age, prior history of AF  [4], post-operative withdrawal of beta-blocker or an angiotensin-converting enzyme (ACE) inhibitor, chronic obstructive lung disease predicting post-operative AF [5]. P-wave duration, increase in P wave dispersion [6], postoperative low cardiac output, preoperative larger left atrium (LA) and LA appendage area, a lower LA ejection fraction, post-bypass atrial systolic dysfunction and abnormal relaxation of the left ventricle, higher preoperative heart rate, increased pulmonary capillary wedge pressure [7], also predicts postoperative AF. It has been implicated that endogenously released adenosine has a role for triggering early (< 48 hr) post-CABG AF [8]. Another issue that received attention was the role of on-pump or off-pump bypass surgery, wherein no significant influence of either on the incidence of postoperative AF could be definitively established [9]. There are two opinions about the change in the autonomic tone brought about by dissection of the anterior epicardial fat or the aortic fat pad influencing the incidence of post CABG AF [10].

It is crucial to identify who would be at risk of developing AF, post CABG surgery, so as to institute appropriate preventive measures. Pharmacological approach has been widely used wherein preoperative initiation of beta-blocker therapy like oral metoprolol and continuation of the same postoperatively has been very effective in preventing AF [11]. Amiodarone, sotalol and propafenone have also been studied. Atrial Fibrillation Suppression Trial (AFIST) [12] showed that oral amiodarone prophylaxis in combination with beta-blockers prevents atrial fibrillation and reduces the risk of cerebrovascular accidents. Similarly in patients with chronic obstructive pulmonary disease, where beta-blockers would be contraindicated, early prophylactic amiodarone was found to reduce AF incidence [13]. Amiodarone and sotalol were found to have similar efficacy and safety in reducing postoperative AF. However, in patients undergoing more complex surgery, postoperative AF occurred more frequently with sotalol than with amiodarone [14].

Based on the mechanism of postoperative AF, it seems likely that overcoming the slow atrial conduction with reduction in the dispersion of atrial refractoriness and suppression of the atrial ectopy should prevent AF. With these considerations atrial overdrive pacing to prevent post CABG AF has been evaluated with a number of randomized, controlled trials. These trials compared controls with either single site atrial pacing i.e. right atrial pacing (RAP) or left atrial pacing (LAP) and biatrial pacing (BAP) or only RAP and BAP or only LAP and BAP. Bachmann’s bundle pacing as an alternative to two sites, biatrial pacing has also been studied. Different pacing modalities (AAT, AAI, DDD, atrial overdrive algorithms) and different stimulation rates are initiated after the surgery and continued for a period of 3-4 days. Earlier studies with pacing highlighted the limitations including sensing and capture problems, increase in atrial ectopy with demand (AAI) pacing and reported no advantage with pacing. However, most recent studies seem to favor prophylactic biatrial pacing to reduce the incidence of post CABG AF [15]. Two studies, one by Goette et al [16] and the other, AFIST II [17] show no impact on post-operative AF. Goette et al studied the role of Bachmann's bundle (BB) pacing, and required early termination of pacing in 16% of patients because of rise in thresholds. The AFIST II evaluated the prophylactic use of a hybrid intravenous and oral amiodarone regimen, atrial septal pacing, or both strategies in post-cardiothoracic surgery (inclusive of valve surgeries) patients. A hybrid intravenous and oral amiodarone regimen was effective at decreasing the incidence of post-operative AF, but atrial septal pacing was found to be ineffective. However, patients receiving both amiodarone and pacing had significantly lower AF rates than those receiving placebo with or without pacing.

The present study involving 120 patients by Massoud E. et al [18] in the present issue is one more study evaluating the role of biatrial pacing in prevention of AF after CABG surgery. This is a prospective, randomized, double blinded-placebo controlled study comparing biatrial pacing with LA pacing and no pacing. The baseline characteristics especially age, LV function, use of beta-blockers, perfusion time and cross-clamp time were well matched in the three groups. Atrial fibrillation occurred in 17.5% patients with bi-atrial pacing, compared to 30% in the LA pacing (p=0.04) and 45% in the control group (p=0.02). Consequently the duration of stay in the intensive care unit and the total hospital stay was significantly reduced in the biatrial-pacing group. The complications were no different between the groups. The time to occurrence and duration of AF was also similar in the three groups. They conclude two sites, biatrial pacing as well tolerated and effective in preventing AF and thereby shorten hospital stay.

It seems use of oral beta-blockers preoperatively and continuing the same postoperatively definitely prevents AF occurrences in the post-operative patients. Amiodarone therapy is especially rewarding when a hybrid intravenous and oral loading regime is employed as suggested by AFIST II trial. A meta-analysis of smaller pacing studies support the role of biatrial pacing. Few questions remain unanswered. Is pacing a cost-effective strategy; should it be prophylactically used in all patients; is there at all a role of single chamber (RA or LA) pacing and single site biatrial pacing (Bachmann bundle or atrial septal pacing)? A larger, multi-center study with a cost effective analysis, over and above the use of beta-blocker and amiodarone is likely to settle the role of prophylactic pacing. Till such time we need to pre-select the high-risk group and offer biatrial pacing.
